# Diagnosis of cardiac surgery-associated acute kidney injury: differential roles of creatinine, chitinase 3-like protein 1 and neutrophil gelatinase-associated lipocalin: a prospective cohort study

**DOI:** 10.1186/s13613-017-0251-z

**Published:** 2017-03-01

**Authors:** Jorien De Loor, Ingrid Herck, Katrien Francois, Astrid Van Wesemael, Lieve Nuytinck, Evelyne Meyer, Eric A. J. Hoste

**Affiliations:** 1 Laboratory of Biochemistry, Department of Pharmacology, Toxicology and Biochemistry, Faculty of Veterinary Medicine, Ghent University, Salisburylaan 133, 9820 Merelbeke, Belgium; 2 Division of Intensive Care, Department of Internal Medicine, Ghent University Hospital, Faculty of Medicine and Health Sciences, Ghent University, De Pintelaan 185, 9000 Ghent, Belgium; 3 Division of Cardiac Surgery, Department of Surgery, Ghent University Hospital, Faculty of Medicine and Health Sciences, Ghent University, De Pintelaan 185, 9000 Ghent, Belgium; 4 Department of Anaesthesiology, General Hospital Sint-Lucas Ghent, Groenebriel 1, 9000 Ghent, Belgium; 5 Bimetra – Clinical Research Centre Ghent, Ghent University Hospital, Ghent University, De Pintelaan 185, 9000 Ghent, Belgium; 6 Research Foundation – Flanders, Egmontstraat 5, 1000 Brussels, Belgium

**Keywords:** Acute kidney injury, Biological markers, Chitinase, Cardiac surgery, Lipocalins

## Abstract

**Background:**

A common and serious complication of cardiac surgery prompting early detection and intervention is cardiac surgery-associated acute kidney injury (CSA-AKI). Urinary chitinase 3-like protein 1 (UCHI3L1) was found to predict AKI associated with critical illness in adults. Our aims were therefore to evaluate whether UCHI3L1 can also be used to predict AKI associated with elective cardiac surgery in adults, and to compare this predictive ability with that of urinary neutrophil gelatinase-associated lipocalin (UNGAL), more frequently assessed early serum creatinine (SCr) measurements, and various two-biomarker panels.

**Methods:**

This was a single-centre prospective cohort study at the eight-bed cardiac surgery ICU of Ghent University Hospital. AKI was diagnosed and classified according to the Kidney Disease|Improving Global Outcomes definitions for the diagnosis and staging of AKI, which are based on SCr and urine output (UO). Of the 211 enrolled elective cardiac surgery patients, we included 203 patients who had no AKI pre-operatively and at time of post-operative ICU admission (t1) in the primary endpoint analysis (i.e. AKI stage ≥1 within 48 h after t1), while 210 patients without AKI stage ≥2 pre-operatively and at t1 were included in the secondary endpoint analysis (i.e. AKI stage ≥2 within 12 h after t1). Systemic and/or urine concentrations of Cr, CHI3L1 and NGAL were measured more frequently than SCr in routine early post-operative ICU practice. UO was monitored hourly in the ICU.

**Results:**

Within 48 h after t1, 46.8% of the patients had developed AKI (70.5% stage 1, 20.0% stage 2 and 9.5% stage 3). In the early post-operative period, only SCr was a good predictor of AKI within 48 h after t1 (primary endpoint). SCHI3L1 combined with either UCHI3L1 or UNGAL was a good predictor of AKI stage ≥2 within 12 h after t1 (secondary endpoint). However, SCr and its absolute difference from pre-operative to early measures after surgery outperformed these combinations.

**Conclusions:**

We found that more frequent assessment of the functional biomarker SCr in the early post-operative ICU period (first 4 h) after elective cardiac surgery in adult patients had good to excellent predictive value for CSA-AKI, indicating that routine SCr assessment must become more frequent in order to detect AKI more early. This performance was in contrast with the inadequate predictive value of the urinary renal stress or damage biomarkers UCHI3L1 and UNGAL.

**Electronic supplementary material:**

The online version of this article (doi:10.1186/s13613-017-0251-z) contains supplementary material, which is available to authorized users.

## Background

Cardiac surgery-associated acute kidney injury (CSA-AKI) is a common and serious complication of cardiac surgery [1]. Depending on its severity and differences in both baseline characteristics and type of cardiac surgical procedure, the range of incidence of CSA-AKI is between 3.0 and 50.0% when applying Kidney Disease|Improving Global Outcomes (KDIGO)-like criteria [2–6]. CSA-AKI treated with renal replacement therapy (RRT) presents post-operatively in 2.0–6.0% of cardiac surgery patients [5, 7, 8], of which 1 out of 2 die in hospital [5]. Importantly, also when the injury is mild, CSA-AKI is independently associated with significant effects on early [i.e. hospital or 30-day (d)] mortality [9, 10].

Risk for CSA-AKI is increased by the presence of established pre-operative [e.g. increased serum creatinine (SCr)] and peri-operative (e.g. low cardiac output) factors that increase susceptibility to AKI [11, 12]. Moreover, potentially important modifiable intra-operative susceptibilities are the type of cardiac surgical technique and, if cardiopulmonary bypass [CPB; synonym: extracorporeal circulation (ECC)] is used, the characteristics of the perfusion technique [13]. Early CSA-AKI occurs within 7 d after the cardiac surgical procedure and is directly related to it [14]. CSA-AKI more than 1 week but within 30 d after the cardiac surgical procedure is mainly related to another exposure that presents as a complication of the cardiac surgical procedure (e.g. sepsis) [14].

Only recently the term ‘acute kidney stress’ (AKS) was proposed to describe the pre-injury phase that leads to AKI [15]. Biomarkers, or ‘measurable and quantifiable biological parameters’ [16], that respond to AKS open new horizons in regard to the prediction and early detection of emerging AKI. These biomarkers serve as surrogate measurements, estimating renal cell perfusion, function or metabolism [15]. As regulator of the iron metabolism, neutrophil gelatinase-associated lipocalin (NGAL) measured in blood or urine represents a way to monitor initial damage [17], which was confirmed in more than 7000 paediatric and adult patients who underwent cardiac surgery [18–20]. Recently, our group demonstrated the role of urinary chitinase 3-like protein 1 (UCHI3L1) as predictor of AKI in an adult general intensive care unit (ICU) cohort, with a performance similar to that of UNGAL [21].

Higher systemic concentrations of CHI3L1 have been independently associated with the presence of coronary artery disease, seeming a quantitative indicator of disease progression as well [22]. Additionally, it was shown that in patients with type 1 and type 2 diabetes mellitus higher systemic concentrations of CHI3L1 were associated with progressing vascular damage in the kidneys, as assessed by the level of albuminuria [22]. It seems plausible that low-grade inflammation and endothelial dysfunction progressing to micro- and macrovascular complications account for higher systemic concentrations of CHI3L1. Besides a marker of chronic inflammation, systemic CHI3L1 is a marker of acute inflammation as well [23]. Cardiac surgery and the use of CPB have a critical role in inducing the systemic inflammatory response syndrome leading to CSA-AKI. Consequently, SCHI3L1 can be proposed as a potential AKI risk factor.

The objectives of this study were:

- To evaluate whether UCHI3L1 can be used to predict occurrence of AKI in adult patients who underwent elective cardiac surgery,
- To compare this predictive ability with that of UNGAL, a well-known biomarker of tubular damage, and that of more frequently assessed early measurements of SCr, a routine biomarker of kidney dysfunction,
- To evaluate whether combining either two urinary renal stress or damage biomarkers, or a systemic kidney dysfunction biomarker and a urinary renal stress or damage biomarker can improve the predictive ability for CSA-AKI,
- To evaluate whether combining SCHI3L1 with either a systemic kidney dysfunction biomarker or a urinary renal stress or damage biomarker can improve the predictive ability for CSA-AKI.

## Methods

This research is reported according to the STrengthening the Reporting of OBservational studies in Epidemiology (STROBE) Statement (Additional file 1: Fig. S1) [24].

### Defining AKI

AKI was diagnosed and classified according to the KDIGO definitions for the diagnosis and staging of AKI, which are based on SCr and urine output (UO) (Additional file 2: Fig. S2) [25].

### Study patients

**Fig. 1:**
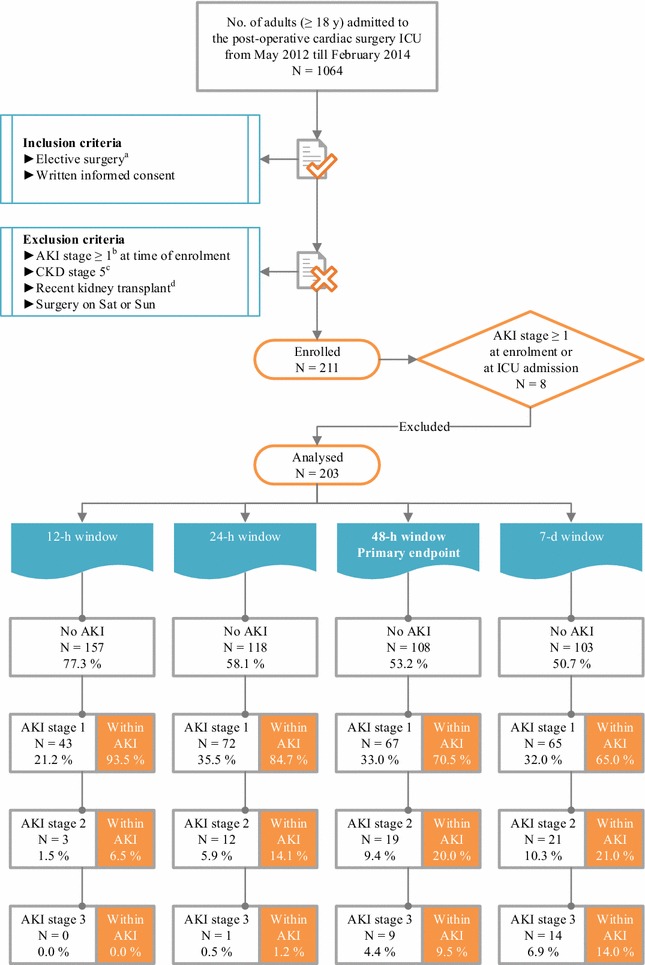
Flow diagram of patient enrolment and primary endpoint analysis. ^a^Planned ≥4 h in advance. ^b^KDIGO definitions for the diagnosis and staging of AKI, which are based on SCr and UO [25]. ^c^KDOQI definitions for the diagnosis and staging of CKD [40]. ^d^≤3 mo before. *AKI* acute kidney injury, *CKD* chronic kidney disease, *d* day, *h* hour, *ICU* intensive care unit, *KDIGO* Kidney Disease|Improving Global Outcomes, *KDOQI* Kidney Disease Outcomes Quality Initiative, *mo* month, *No.* number, *Sat* Saturday, *SCr* serum creatinine, *Sun* Sunday, *UO* urine output, *y* year

In this single-centre cohort study, we prospectively enrolled patients who were admitted to the eight-bed cardiac surgery ICU of Ghent University Hospital from May 2012 till February 2014. The inclusion and exclusion criteria of the study are incorporated in the flow diagram in Fig. 1.

### Primary endpoint

The primary endpoint of the current study was the development of AKI stage ≥1 within 48 h after t1. Reference SCr, representing baseline SCr, was defined as the lowest SCr value within the last 3 months (mo) prior to enrolment (lowest of history SCr value(s) and pre-operative SCr). In our cohort 30.0% of the patients had only the pre-operative SCr value available. In 49.3% the lowest history SCr was lower than or equal to the pre-operative SCr, while in 20.7% the pre-operative SCr was lower than the lowest history SCr. The details for calculation of UO are outlined by our group [21]. Note that UO was registered hourly in the ICU only.

### Secondary endpoint

The secondary endpoint was AKI stage ≥2 within 12 h after t1.

### Ethics approval and consent to participate

The Ethical Committee of Ghent University Hospital approved this study (Belgian Registration Number of the study: B670201213147). All patients or their legally authorized representatives provided written informed consent. We respected the Declaration of Helsinki and the Good Clinical Practice Guidelines.

### Prospective sample and data collection

The first collection of blood and urine was after the induction of anaesthesia and before the start of surgery [time 0 (t0) on the day of surgery (d_surgery_)]. The rest of the specimens (*n* = 7) were collected post-operatively, starting at ICU admission (t1) and then at 2 hours (h) (t2), 4 h (t3), 6 h, 12 h, 24 h and 48 h after ICU admission. If the patient was discharged to the Midcare unit before 24 h or 48 h, those samples were collected there. Whenever possible, the routine collection times were followed [at 4 PM on the first post-operative day (d_1post-op_) and at 6 AM on the second post-operative day (d_2post-op_)]. The sample collection times for a fictional patient who underwent surgery in the morning are outlined on the timeline in Additional file 3: Fig. S3A, while those for a fictional patient who underwent surgery in the afternoon are outlined on the timeline in Additional file 4: Fig. S3B.

These paired blood and urine samples were collected by standard methods and centrifuged by standard protocols, as described previously by our group [21]. Serum and urine supernatants were stored at −80 °C and thawed at room temperature immediately prior to analysis. Clinical data needed to complete the individual clinical research files (Additional file 5: Table S1) were extracted from the hospital records by study coordinators. Note that Additional file 5 contains Tables S1, S2, S3, S4A, B, C, D and the legends of Figures S1, S2, S3A, B, S4, S5, S6. Samples were anonymized as were clinical data. All technicians were blinded to clinical data.

### Biomarker analysis and single-biomarker diagnostic test possibilities

The CHI3L1 analysis was performed in-house. We measured the concentration of CHI3L1 by a sandwich enzyme-linked immunosorbent assay (ELISA) technique (DC3L10, R&D Systems, Minneapolis, MN, USA). Analyses performed externally were Cr and UNGAL. The Cobas c502 measured the concentration of Cr by a kinetic rate-blanked Jaffé assay (commercial reagents, Roche Diagnostics, Basel, Switzerland), whereas the Modular P measured the concentration of UNGAL by a particle-enhanced turbidimetric immunoassay (ST001-3CA, BioPorto, Hellerup, Denmark). All details were recently described [21], except for the standard sample dilution scheme used in the CHI3L1 ELISA, which is presented in Additional file 5: Table S2. For blood samples that were collected at routine collection times, a SCr concentration was already available in the hospital records. Based on the temporal relationship of the predictive value of UNGAL for CSA-AKI [19], we measured this biomarker at t1 and t3 only.

Besides UCHI3L1 and UNGAL, we also evaluated UCHI3L1 and UNGAL corrected for urine dilution by using the ratio to UCr as diagnostic test. Besides SCr, we also evaluated ΔSCr_tx-t0_ as diagnostic test, representing the absolute change in SCr between SCr_tx_ and SCr_t0_. The most recent SCr value recorded prior to surgery was considered as SCr_t0_.

### Defining acute tubular damage and subclinical AKI

Following the recommendations of de Geus et al., acute tubular damage was defined as a CSA-NGAL score of 2 or greater; either as UNGAL_t1_ or UNGAL_t3_ ≥ 150 ng/ml or as ∆UNGAL_t3-t1_ > 100 ng/ml with UNGAL_t3_ ≥ 125 ng/ml [26]. Subclinical AKI was defined when there was acute tubular damage (according to the ‘de Geus criteria’) and absence of AKI according to the KDIGO definition.

### Defining good and excellent biomarkers

An area under the receiver operating characteristics curve (AUC-ROC) of 0.750 or greater was considered to represent a good biomarker, whereas an AUC-ROC of 0.900 or greater was considered to represent an excellent biomarker [27].

### Statistical analysis

The principal statistical analysis was based on comparison of the AUC-ROCs of UCHI3L1 with those of UNGAL, more frequently assessed early measurements of SCr, and various two-biomarker panels for predicting both defined endpoints. It was performed in MedCalc 15.2.1 (MedCalc Software, Oostende, Belgium). The unpaired comparison of a variable between two independent samples was done in SPSS 22 (IBM, Armonk, NY, USA). Categorical variables were analysed with Fisher’s exact or the Chi-square test, and continuous variables with the nonparametric Mann–Whitney *U* test. Additionally, we calculated the 95% confidence interval (CI) for a proportion using the Wilson procedure without a correction for continuity [28, 29]. For all analyses, two-sided *P* values <0.05 were considered statistically significant. All details and a description of how the biomarkers were introduced into the statistical programs are provided in our previous work [21].

## Results

### Incidence of the primary endpoint, characteristics of the patients and procedures, and short-term patient outcomes

**Fig. 2:**
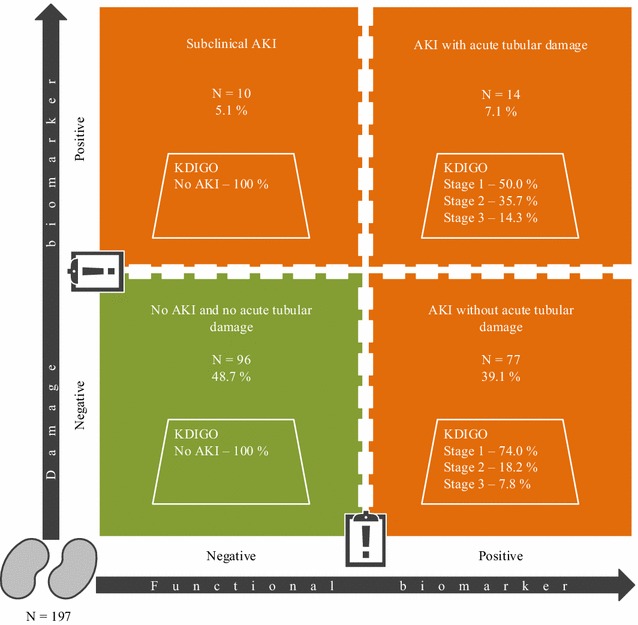
Combining functional and damage biomarkers simultaneously to delineate the spectrum of AKI. Patients of the primary analysis cohort (*n* = 203) who had no UNGAL_t1_ or UNGAL_t3_ concentration available were excluded. Missing UNGAL_t1_ occurred in 5 patients, and missing UNGAL_t3_ in 1 patient, resulting in a total of 197 patients. Following the recommendations of de Geus et al., acute tubular damage was defined as a CSA-NGAL score of 2 or greater; either as UNGAL_t1_ or UNGAL_t3_ ≥ 150 ng/ml or as ∆UNGAL_t3−t1_ > 100 ng/ml with UNGAL_t3_ ≥ 125 ng/ml [26]. Subclinical AKI was defined when there was acute tubular damage (according to the ‘de Geus criteria’) and absence of AKI according to the KDIGO definition. In this way 84.6% of AKI in our specific cohort (i.e. 77/[77 + 14]) was classified as AKI without acute tubular damage. Subclinical AKI, which was missed by KDIGO, occurred in 5.1% of the patients. *AKI* acute kidney injury, *CSA* cardiac surgery-associated, *d* day, *KDIGO* Kidney Disease|Improving Global Outcomes, *t1* time of intensive care unit admission, *t3* 4 h after intensive care unit admission, *UNGAL* urinary neutrophil gelatinase-associated lipocalin

The flow of patients during the study is illustrated in Fig. 1. The initial enrolment cohort consisted of 211 patients. In the primary endpoint analysis, 8 patients who already had AKI stage ≥1 at t0 (*n* = 3) or t1 were excluded, resulting in a total number of 203 patients for analysis. Within 48 h after t1, 95 patients (46.8%) had developed AKI: 67 (70.5%) were classified as stage 1, 19 (20.0%) as stage 2 and 9 (9.5%) as stage 3. Three patients received RRT starting on d_2post-op_. Duration of RRT was 3 d in 2 patients and 14 d in 1 patient. The flow of patients over different diagnostic windows for AKI is illustrated in Fig. 1. The limited extent to which the UO criterion identified AKI patients is illustrated in Additional file 6: Fig. S4. Median time to AKI diagnosis was 12.2 h [interquartile range (IQR) 7.2–18.4 h]. Subclinical AKI, which was missed by KDIGO, occurred in 5.1% of the patients (Fig. 2).

Table 1 summarizes the characteristics of the patients and procedures at baseline. Compared with patients without AKI, patients who developed AKI within 48 h after t1 were older; had a higher body mass index (BMI); a lower eGFR; a higher prevalence of diabetes mellitus (DM) type 2; a higher predicted operative mortality, which was estimated by the simple additive European system for cardiac operative risk evaluation (EuroSCORE); more combined surgical procedures; and a higher prevalence of diuretic treatment at home.

**Table 1:** Characteristics of the patients and procedures at baseline, as well as short-term patient outcomes

No. (%) [95% CI]	All patients 203 (100) [98.1–100]	AKI stage ≥1^a^ within 48 h 95 (46.8) [40.1–53.7]	No AKI^a^ within 48 h 108 (53.2) [46.3–59.9]	*P* value
*Characteristic*				
Male sex—no. (%) [95% CI]	133 (65.5) [58.7–71.7]	65 (68.4) [58.5–76.9]	68 (63.0) [53.6–71.5]	0.461
White race—no. (%) [95% CI]	202 (99.5) [97.3–99.9]	95 (100) [96.1–100]	107 (99.1) [94.9–99.8]	1.000
Age^b^ (IQR)—years	70.0 (61.0–76.0)	74.0 (65.0–80.0)	67.0 (58.0–75.0)	<0.001
BMI (IQR)	27 (24–29)	27 (25–31)	26 (23–29)	0.004
*Medical history*				
Reference renal function (IQR)				
SCr—mg/dl	0.90 (0.75–1.05)	0.99 (0.81–1.16)	0.82 (0.70–0.95)	<0.001
eGFR_CKD-EPI_—ml/min/1.73 m^2^	82 (64–94)	73 (54–85)	87 (77–97)	<0.001
DM—no. (%) [95% CI]				0.025
Type 1	2 (1.0) [0.3–3.5]	2 (2.1) [0.6–7.4]	0 (0.0) [0.0–3.4]	
Type 2	46 (22.7) [17.4–28.9]	28 (29.5) [21.2–39.3]	18 (16.7) [10.8–24.8]	
No DM	155 (76.4) [70.1–81.7]	65 (68.4) [58.5–76.9]	90 (83.3) [75.2–89.2]	
Heart failure—no. (%) [95% CI]				0.252
NYHA class I	145 (71.4) [64.9–77.2]	64 (67.4) [57.4–76.0]	81 (75.0) [66.1–82.2]	
NYHA class II	36 (17.7) [13.1–23.6]	17 (17.9) [11.5–26.8]	19 (17.6) [11.6–25.8]	
NYHA class III	20 (9.9) [6.5–14.7]	12 (12.6) [7.4–20.8]	8 (7.4) [3.8–13.9]	
NYHA class IV	2 (1.0) [0.3–3.5]	2 (2.1) [0.6–7.4]	0 (0.0) [0.0–3.4]	
*Clinical examination*				
Blood pressure (IQR)—mm Hg				
Systolic	134 (122–149)	132 (122–150)	134 (120–149)	0.704
Diastolic	72 (64–78)	72 (63–78)	71 (66–78)	0.619
Mean	93 (85–101)	92 (84–102)	93 (86–100)	0.734
Heart rhythm—no. (%) [95% CI]				0.074
Atrial fibrillation	16 (7.9) [4.9–12.4]	11 (11.6) [6.6–19.6]	5 (4.6) [2.0–10.4]	
Normal sinus rhythm	187 (92.1) [87.6–95.1]	84 (88.4) [80.4–93.4]	103 (95.4) [89.6–98.0]	
Heart rate in normal sinus rhythm (IQR)—bpm (*N* = 187)	69 (61–79)	70 (62–81)	69 (60–76)	0.143
Distribution of ejection fraction—no. (%) [95% CI]				0.531
≤20%	5 (2.5) [1.1–5.6]	4 (4.2) [1.6–10.3]	1 (0.9) [0.2–5.1]	
21–30%	3 (1.5) [0.5–4.3]	2 (2.1) [0.6–7.4]	1 (0.9) [0.2–5.1]	
31–50%	33 (16.3) [11.8–22.0]	16 (16.8) [10.6–25.6]	17 (15.7) [10.1–23.8]	
>50%	119 (58.6) [51.7–65.2]	55 (57.9) [47.8–67.3]	64 (59.3) [49.8–68.1]	
*Index surgical procedure*				
EuroSCORE (IQR)	5 (3–8)	6 (4–9)	5 (2–7)	0.004
Type of cardiac surgical procedure—no. (%) [95% CI]				0.005
Isolated CABG	93 (45.8) [39.1–52.7]	34 (35.8) [26.9–45.8]	59 (54.6) [45.2–63.7]	
Isolated valve repair or replacement	55 (27.1) [21.4–33.6]	28 (29.5) [21.2–39.3]	27 (25.0) [17.8–33.9]	
CABG and valve repair or replacement	34 (16.7) [12.2–22.5]	25 (26.3) [18.5–36.0]	9 (8.3) [4.4–15.1]	
Aortic root	12 (5.9) [3.4–10.0]	5 (5.3) [2.3–11.7]	7 (6.5) [3.2–12.8]	
Other	9 (4.4) [2.3–8.2]	3 (3.2) [1.1–8.9]	6 (5.6) [2.6–11.6]	
ECC—no. (%) [95% CI]				0.516
Yes	179 (88.2) [83.0–91.9]	82 (86.3) [78.0–91.8]	97 (89.8) [82.7–94.2]	
No	24 (11.8) [8.1–17.0]	13 (13.7) [8.2–22.0]	11 (10.2) [5.8–17.3]	
Duration of ECC (IQR)—min *N* = 179	91.5 (70.8–123.3)	92.0 (70.3–131.3)	91.5 (70.3–117.8)	0.527
Priming volume of ECC pump (IQR)—ml *N* = 179	1300 (1200–1500)	1300 (1200–1500)	1300 (1150–1400)	0.246
Duration of aortic clamp during ECC (IQR)—min *N* = 179	56.0 (42.8–82.0)	62.0 (45.0–88.0)	55.0 (40.5–74.5)	0.174
Duration of ischaemia during ECC (IQR)—min *N* = 179	54.0 (39.0–78.0)	61.0 (43.0–81.0)	52.0 (37.8–70.5)	0.164
Duration of surgery (IQR)—h	4.6 (3.9–5.2)	4.7 (3.9–5.3)	4.5 (3.9–5.1)	0.196
IABP peri-operatively—no. (%) [95% CI]	7 (3.4) [1.7–6.9]	2 (2.1) [0.6–7.4]	5 (4.6) [2.0–10.4]	0.452
*Medication—no. (%) [95% CI]*				
Statins	127 (62.6) [55.7–68.9]	63 (66.3) [56.3–75.0]	64 (59.3) [49.8–68.1]	0.313
ACE inhibitors	56 (27.6) [21.9–34.1]	28 (29.5) [21.2–39.3]	28 (25.9) [18.6–34.9]	0.638
ARBs	7 (3.4) [1.7–6.9]	6 (6.3) [2.9–13.1]	1 (0.9) [0.2–5.1]	0.052
Diuretics	51 (25.1) [19.7–31.5]	36 (37.9) [28.8–47.9]	15 (13.9) [8.6–21.7]	<0.001
NSAIDs	4 (2.0) [0.8–5.0]	1 (1.1) [0.2–5.7]	3 (2.8) [0.9–7.9]	0.624
Corticosteroids	13 (6.4) [3.8–10.6]	8 (8.4) [4.3–15.7]	5 (4.6) [2.0–10.4]	0.390
Tacrolimus	0 (0.0) [0.0–1.9]	0 (0.0) [0.0–3.9]	0 (0.0) [0.0–3.4]	NA
Cyclosporine	2 (1.0) [0.3–3.5]	2 (2.1) [0.6–7.4]	0 (0.0) [0.0–3.4]	0.218
Aminoglycosides	3 (1.5) [0.5–4.3]	1 (1.1) [0.2–5.7]	2 (1.9) [0.5–6.5]	1.000
Corticosteroids intra-operatively	0 (0.0) [0.0–1.9]	0 (0.0) [0.0–3.9]	0 (0.0) [0.0–3.4]	NA
Iodinated contrast ≤72 h before surgery	37 (18.2) [13.5–24.1]	14 (14.7) [9.0–23.2]	23 (21.3) [14.6–29.9]	0.275
*Outcomes*				
RRT in ICU—no. (%) [95% CI]	3 (1.5) [0.5–4.3]	3 (3.2) [1.1–8.9]	0 (0.0) [0.0–3.4]	0.101
ICU LOS (IQR)—d	1 (1–3)	2 (1–3)	1 (1–2)	<0.001
Hospital LOS (IQR)—d	12 (9–16)	13 (10–20)	10 (9–13)	<0.001

*ACE* angiotensin-converting enzyme, *AKI* acute kidney injury, *ARB* angiotensin-II receptor blocker, *BMI* body mass index, *bpm* beats per minute, *CABG* coronary artery bypass grafting, *CI* confidence interval, *CKD*-*EPI* Chronic Kidney Disease Epidemiology Collaboration, *DM* diabetes mellitus, *ECC* extracorporeal circulation, *eGFR* estimated glomerular filtration rate, *EuroSCORE* European system for cardiac operative risk evaluation, *h* hour, *IABP* intra-aortic balloon pump, *ICU* intensive care unit, *IQR* interquartile range, *KDIGO* Kidney Disease|Improving Global Outcomes, *LOS* length of stay, *min* minute, *no.* number, *NSAID* nonsteroidal anti-inflammatory drug, *NYHA* New York Heart Association, *RRT* renal replacement therapy, *SCr* serum creatinine, *UO* urine output

^a^KDIGO definitions for the diagnosis and staging of AKI, which are based on SCr and UO

^b^Determined at the day of surgery

Peri-operative characteristics of the patients and procedures are provided in Additional file 5: Table S3. In comparison with patients without AKI, patients who developed AKI within 48 h after t1 had a higher Sepsis-related Organ Failure Assessment (SOFA) score; a higher white blood cell (WBC) count; a higher serum C-reactive protein (CRP) concentration; a more positive fluid balance; a higher number of plasma units that were transfused; and a higher prevalence of vasopressor, milrinone and diuretic treatment.

AKI patients had a longer ICU and hospital length of stay (LOS) (Table 1).

### Incidence of the secondary endpoint

In the secondary endpoint analysis, 1 patient who already had AKI stage ≥2 at t0 was excluded, resulting in a total number of 210 patients for analysis. Within 12 h after t1, 5 patients (2.4%) had developed AKI stage ≥2: all 5 (100%) were classified as stage 2. The flow of patients over different diagnostic windows for AKI stage ≥2 is illustrated in Additional file 7: Fig. S5.

### Biomarker performances for prediction of the primary endpoint

**Fig. 3:**
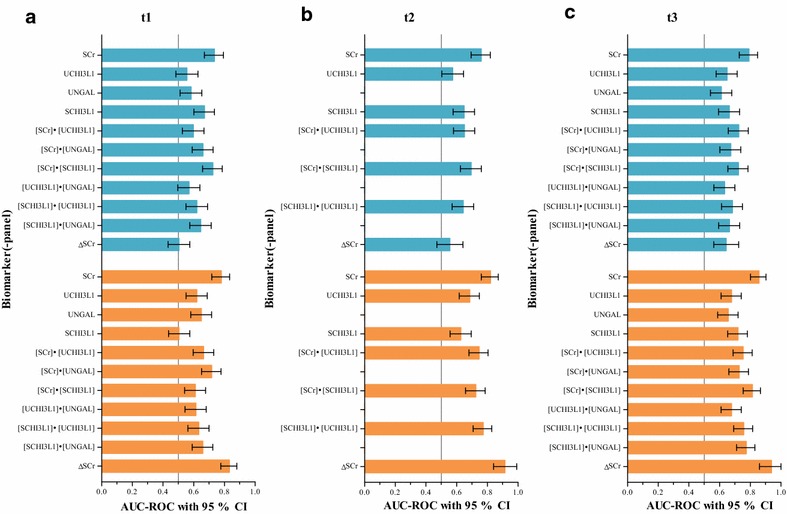
Biomarker performances for prediction of AKI. Blue boxes correspond with the primary endpoint (i.e. AKI stage ≥1 within 48 h after t1), orange boxes with the secondary endpoint (i.e. AKI stage ≥2 within 12 h after t1). ΔSCr represents ΔSCr_tx-t0_, which is the absolute change in SCr between SCr_tx_ and SCr_t0_. Biomarkers were measured **a** at ICU admission (t1), **b** 2 h after ICU admission (t2) and **c** 4 h after ICU admission (t3). *AKI* acute kidney injury, *h* hour, *ICU* intensive care unit, *SCHI3L1* serum chitinase 3-like protein 1, *SCr* serum creatinine, *UCHI3L1* urinary chitinase 3-like protein 1, *UNGAL* urinary neutrophil gelatinase-associated lipocalin

In the early post-operative period, only the functional biomarker SCr was a good predictor of AKI within 48 h after t1, showing the highest AUC-ROC at t3 (0.792; 95% CI 0.728–0.847; Fig. 3). The information in Fig. 3 is summarized in numerical format in Additional file 5: Tables S4B, C and D, including also the performances of the urinary renal stress or damage biomarkers corrected for urine dilution by using the ratio to UCr. Table S4A reports the performances of the biomarkers measured at t0.

### Biomarker performances for prediction of the secondary endpoint

In the early post-operative period, SCHI3L1 combined with a urinary renal stress or damage biomarker, either UCHI3L1 or UNGAL, was a good predictor of AKI stage ≥2 within 12 h after t1, showing the highest AUC-ROC at t2 when combined with UCHI3L1 (0.773; 95% CI 0.708–0.829) and at t3 when combined with UNGAL (0.774; 95% CI 0.710–0.830). However, the functional biomarkers SCr and ΔSCr_tx-t0_ outperformed these combinations with good to excellent performances, showing the highest AUC-ROC at t3 (0.857 for SCr; 95% CI 0.801–0.902; 0.938 for ΔSCr_tx−t0_; 95% CI 0.860–1.000; Fig. 3). The information in Fig. 3 is summarized in numerical format in Additional file 5: Tables S4B, C and D, including also the performances of the urinary renal stress or damage biomarkers corrected for urine dilution by using the ratio to UCr. Table S4A reports the performances of the biomarkers measured at t0.

## Discussion

We found that in adult patients who underwent elective cardiac surgery, UCHI3L1 had inadequate predictive value for CSA-AKI. This was also true for the well-known tubular damage biomarker UNGAL. In contrast, more frequent assessment of the functional biomarker SCr in the early post-operative ICU period (first 4 h) had good to excellent predictive value for CSA-AKI.

Similar to others, our ICU routinely measures SCr at t1 and either around t4 (morning patient) or t5 (afternoon patient) in the early post-operative period. However, with ±50% of AKI diagnosed before t5, of which ±50% before t4, our study highlights the importance of more frequent SCr assessment in the first 4 h. This aids in early AKI diagnosis and could also reveal some cases of rapid reversal of AKI (i.e. ‘complete reversal of AKI by KDIGO criteria within 48 h of AKI onset’ [30]). These findings are in accordance with those of a small retrospective study (*n* = 29) by Maciel et al. [31].

Novel findings of our study are the good to excellent performances of ΔSCr_t1−t0_, ΔSCr_t2−t0_ and ΔSCr_t3−t0_ for the prediction of AKI stage ≥2 within 12 h after t1. In accordance with these results, measurement of very early post-operative SCr changes, either absolute [10] or relative [32, 33], in adult [10, 33] and paediatric [32] cardiac surgery patients has been reported to provide prognostic utility for a subsequent diagnosis of AKI. However, each of these three studies was restricted by some of the following limitations: AKI was not defined according to KDIGO, including oliguric criteria; more severe AKI was not included as endpoint; SCr was less frequently assessed in the early post-operative period; and AUC-ROC analysis was not reported.

The simultaneous utilization of functional and damage biomarkers, as in the CSA-NGAL score [26], permits to delineate the spectrum of AKI [34]. In this way 84.6% of AKI in our specific cohort (i.e. 77/[77 + 14]) was classified as AKI without acute tubular damage. The capacity of the kidneys to increase baseline GFR under physiological or pathological kidney stress is described by the renal functional reserve of the glomerular function (Additional file 8: Fig. S6) [35]. In congestive heart failure, CKD and AKI, utilization of the renal functional reserve of the glomerular function allows to replace (in part) the lost function, maintaining a normal whole-organ baseline GFR until ±50% of the nephrons are nonfunctional. The presence of an intact renal functional reserve of the glomerular function and nephron mass guarantees a low renal frailty or susceptibility [36]. Compared with the general ICU cohort of our previous AKI biomarker study [21], the patients in this study were older (median 70.0 vs. 60.0 y), more often diabetic (median 23.6 vs. 7.2%), had a higher BMI (median 27 vs. 24) and a lower baseline eGFR (median 82 vs. 102 ml/min/1.73 m^2^). We therefore hypothesize that a lower renal functional reserve of the glomerular function and nephron mass in this population caused the low incidence of both subclinical AKI and AKI with acute tubular damage. This can also explain the inadequate performance of the urinary renal stress or damage biomarkers UCHI3L1 and UNGAL for prediction of AKI stage ≥2 within 12 h after t1. Our study is, however, not the first to report inadequate diagnostic ability of UNGAL for moderate to severe CSA-AKI [20]. In their multicentre high-risk cohort with 21% emergent surgeries, 42% diabetic patients and a baseline eGFR of 67 ml/min/1.73 m^2^, Parikh et al. reported an AUC-ROC of 0.67 (standard error (SE) 0.04) for the prediction of AKI defined by receipt of RRT or doubling of SCr [37]. Interestingly, Koyner et al. found an AUC-ROC of 0.88 (95% CI 0.73–0.99) for the prediction of AKI Acute Kidney Injury Network (AKIN) stage 3 in a cohort comparable to ours [38], indicating the importance of the severity of AKI in the predictive ability of UNGAL.

Our study has several limitations. First, it is limited in being a single-centre study in elective cardiac surgery patients. However, baseline characteristics and the incidence of AKI suggest that our population is representative of cardiac surgery ICUs in developed countries. Second, our study is further limited by the relatively low incidence of AKI stage ≥2 within 12 h after t1 (secondary endpoint). Both the fact that we included only elective surgeries and the implemented narrow diagnostic window contribute to this constraint. As such, these biomarker data need to be further validated in larger multicentre prospective studies. Third, we did not measure the panel of G1 cell-cycle arrest biomarkers tissue inhibitor of metalloproteinases 2 (TIMP-2) and insulin-like growth factor-binding protein 7 (IGFBP7) [39], because it was not available at the start of our study.

## Conclusions

In summary, we demonstrated that more frequent assessment of the functional biomarker SCr in the early post-operative ICU period (first 4 h) after elective cardiac surgery in adult patients had good to excellent predictive value for CSA-AKI, indicating that routine SCr assessment must become more frequent in order to detect AKI more early. We found that AKI was predominantly without acute tubular damage and that subclinical AKI was uncommon in this cohort. This may explain the inadequate predictive value for CSA-AKI of the urinary renal stress or damage biomarkers UCHI3L1 and UNGAL. These results need to be further validated in larger multicentre prospective studies.

## Additional files

**Additional file 1: Figure S1.** STROBE statement—checklist of items that should be included in the reports of cohort studies (1).

**Additional file 2: Figure S2.** KDIGO definition and classification of AKI (2).

**Additional file 3: Figure S3A.**Study course and sample collection times in a fictional morning patient.

**Additional file 4: Figure S3B** Study course and sample collection times in a fictional afternoon patient.

**Additional file 5.** **Table S1**: Data collected in the clinical research file. **Table S2**: Dilution of serum and urine samples for the initial measurement of CHI3L1 by ELISA. **Table S3**: Characteristics of the patients and procedures peri-operatively. **Table S4A**: Biomarker performances at t0 for prediction of AKI. **Table S4B**: Biomarker performances at t1 for prediction of AKI. **Table S4C**: Biomarker performances at t2 for prediction of AKI. **Table S4D**: Biomarker performances at t3 for prediction of AKI.

**Additional file 6: Figure S4** Dissociation of the KDIGO definitions for the diagnosis and staging of AKI by SCr and UO.

**Additional file 7: Figure S5.** Flow of patients over different diagnostic windows for AKI stage ≥2.

**Additional file 8: Figure S6.** Renal functional reserve of the glomerular function and functioning nephron mass.

## Abbreviations

CSA-AKI: cardiac surgery-associated acute kidney injury
KDIGO: Kidney Disease|Improving Global Outcomes
RRT: renal replacement therapy
d: day
SCr: serum creatinine
CPB: cardiopulmonary bypass
ECC: extracorporeal circulation
AKS: acute kidney stress
NGAL: neutrophil gelatinase-associated lipocalin
UCHI3L1: urinary chitinase 3-like protein 1
ICU: intensive care unit
STROBE: STrengthening the Reporting of OBservational studies in Epidemiology
UO: urine output
t0: time after the induction of anaesthesia and before the start of surgery
d_surgery_: day of surgery
t1: time of intensive care unit admission
h: hour
t2: 2 h after intensive care unit admission
t3: 4 h after intensive care unit admission
d_1post-op_: first post-operative day
d_2post-op_: second post-operative day
ELISA: enzyme-linked immunosorbent assay
AUC-ROC: area under the receiver operating characteristics curve
mo: month
eGFR: estimated glomerular filtration rate
CKD-EPI: Chronic Kidney Disease Epidemiology Collaboration
CI: confidence interval
IQR: interquartile range
BMI: body mass index
DM: diabetes mellitus
EuroSCORE: European system for cardiac operative risk evaluation
SOFA: Sepsis-related Organ Failure Assessment
WBC: white blood cell
CRP: C-reactive protein
LOS: length of stay
SE: standard error
AKIN: Acute Kidney Injury Network
OR: odds ratio
RIFLE: risk, injury, failure, loss and end-stage
HR: hazard ratio
TIMP-2: tissue inhibitor of metalloproteinases 2
IGFBP7: insulin-like growth factor-binding protein 7
