# Supplementary figures and images for: Diagnosis of cardiac surgery-associated acute kidney injury: differential roles of creatinine, chitinase 3-like protein 1 and neutrophil gelatinase-associated lipocalin: a prospective cohort study
Source: Ann Intensive Care. 2017 Mar 1;7:24. doi: 10.1186/s13613-017-0251-z (PMC5332341; doi:10.1186/s13613-017-0251-z)

## Diagnosis of AKI by KDIGO

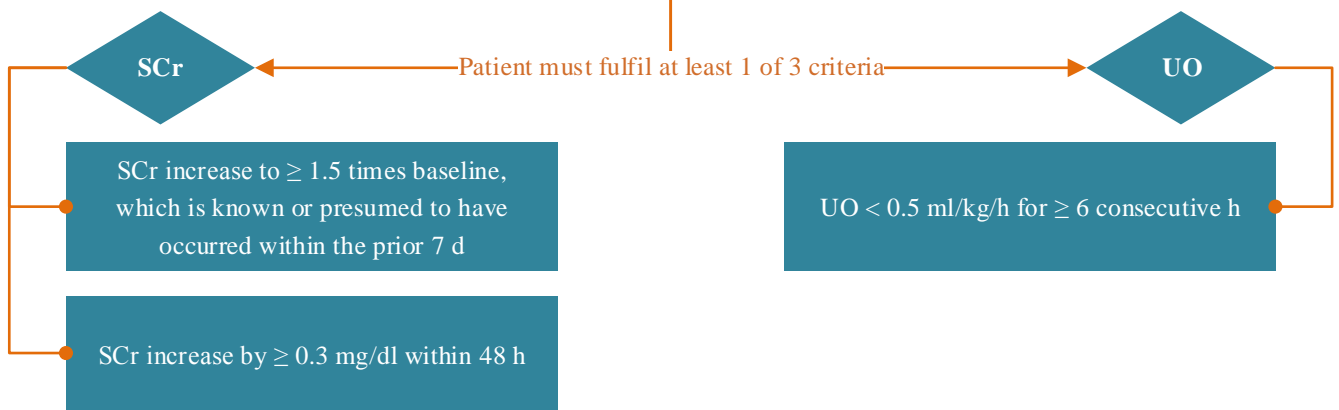

## Staging<sup>a</sup> of AKI by KDIGO

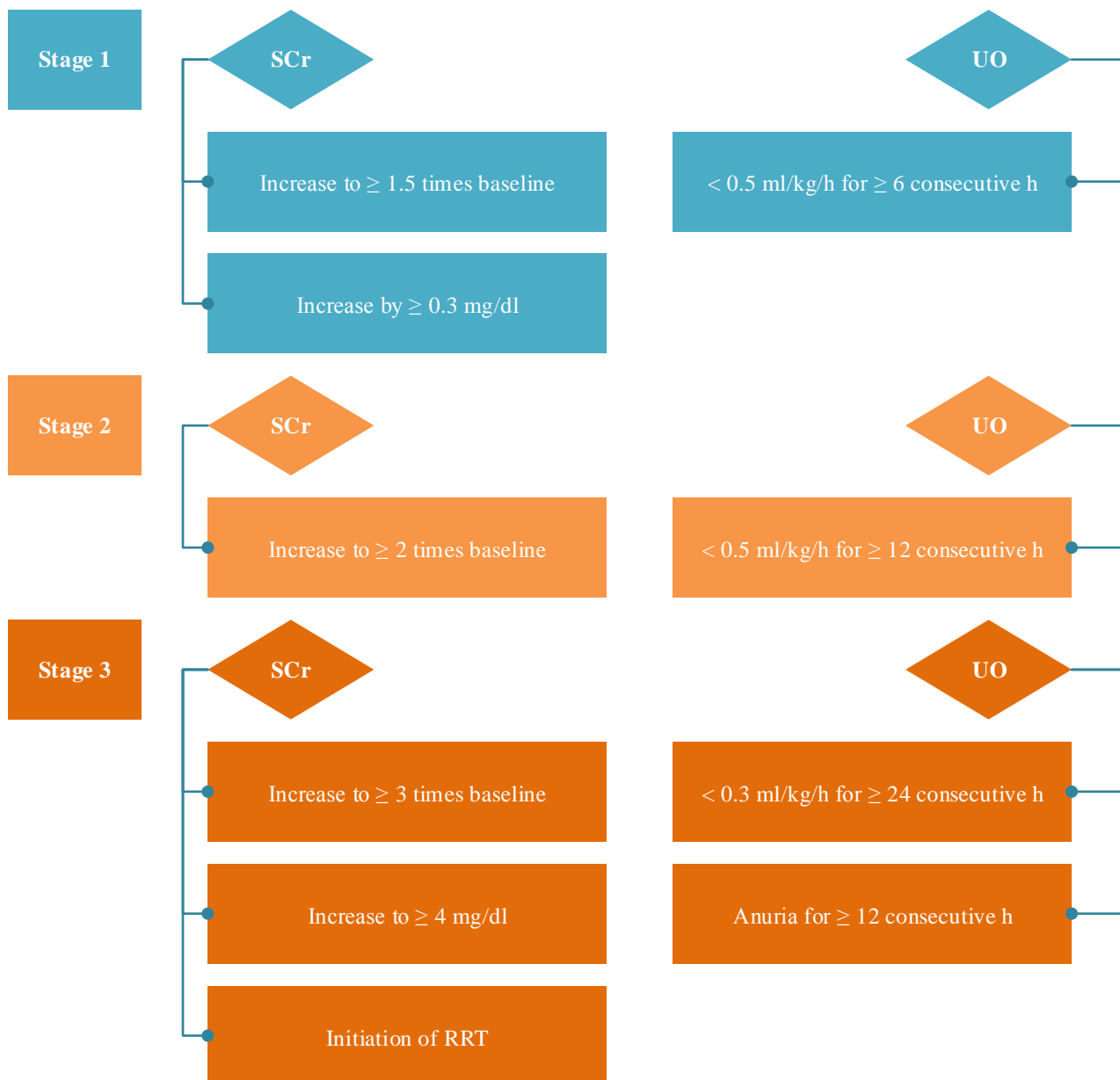

Supplement: Supplementary file 2 — Additional file 2: Figure S2. KDIGO definition and classification of AKI (2). [file 13613_2017_251_MOESM2_ESM.pdf]

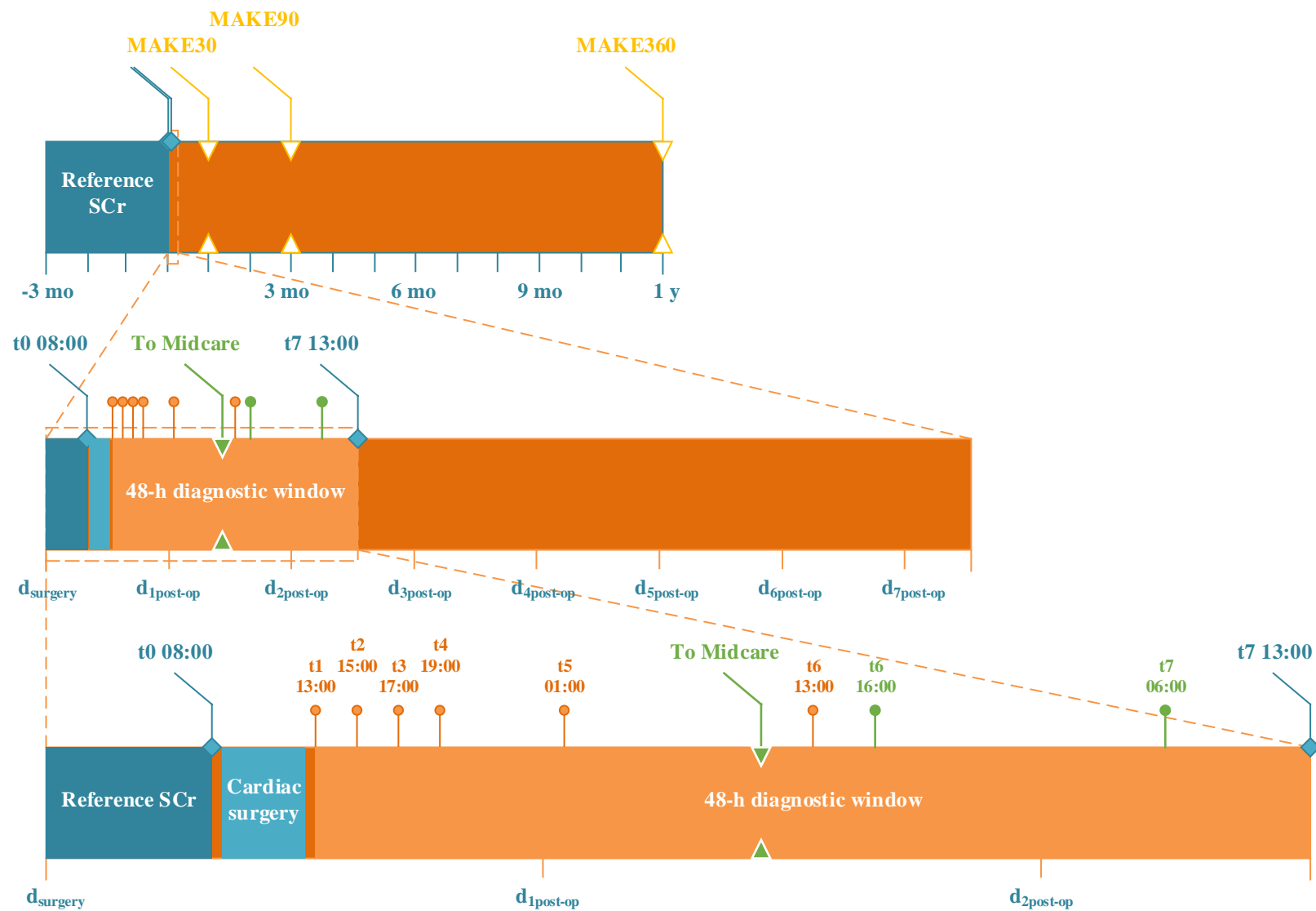

Supplement: Supplementary file 3 — Additional file 3: Figure S3A.Study course and sample collection times in a fictional morning patient. [file 13613_2017_251_MOESM3_ESM.pdf]

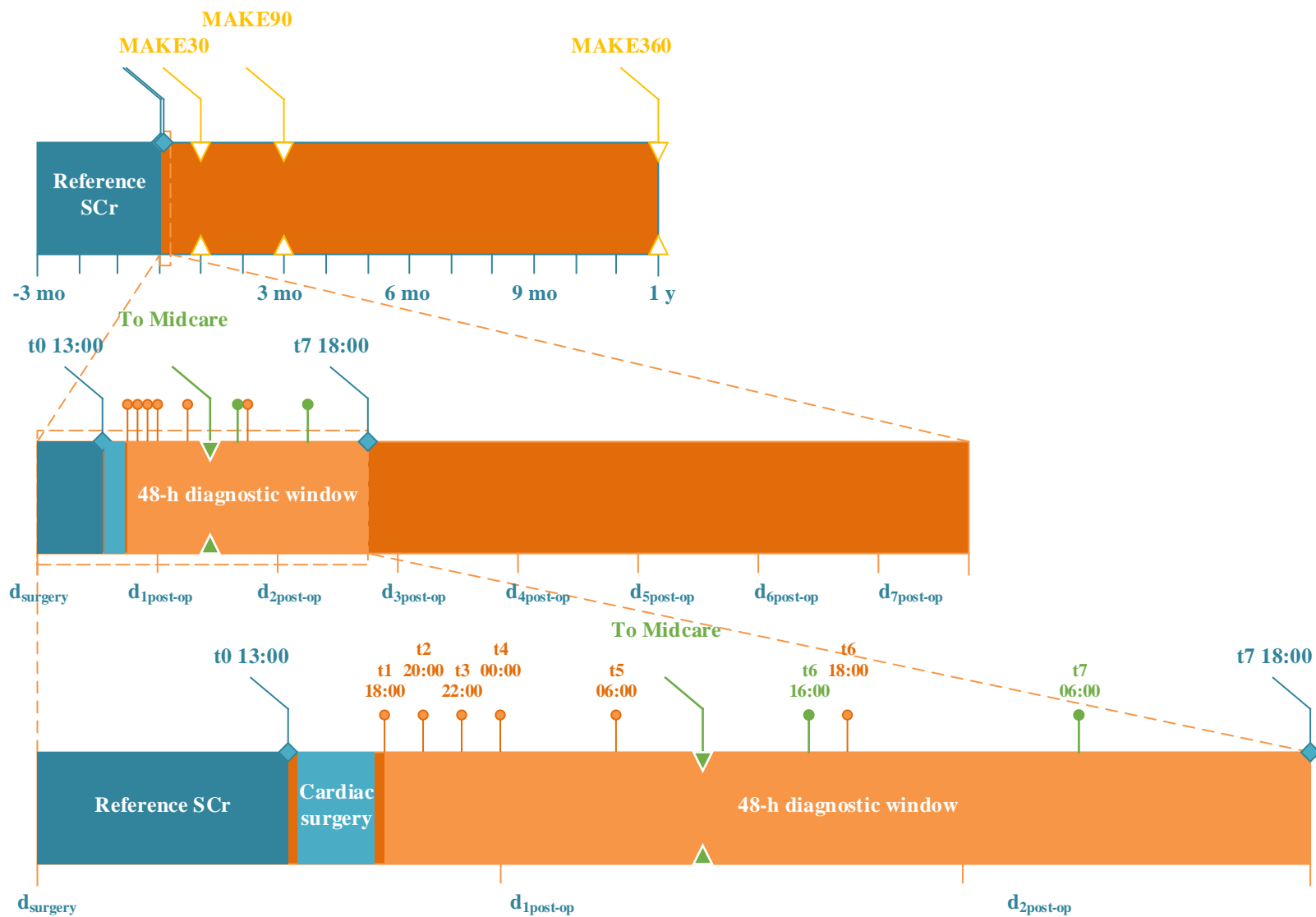

Supplement: Supplementary file 4 — Additional file 4: Figure S3B Study course and sample collection times in a fictional afternoon patient. [file 13613_2017_251_MOESM4_ESM.pdf]

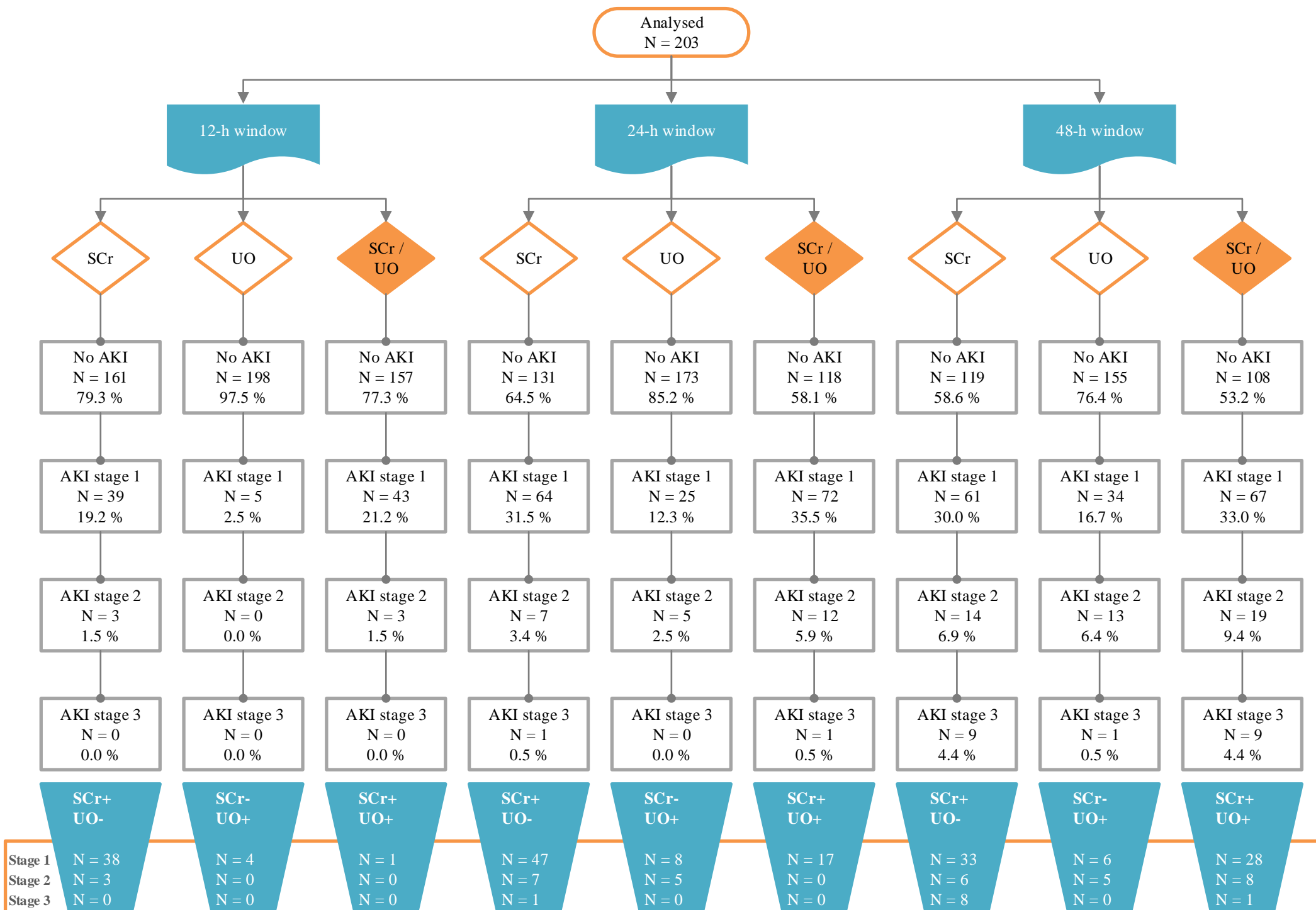

Supplement: Supplementary file 6 — Additional file 6: Figure S4 Dissociation of the KDIGO definitions for the diagnosis and staging of AKI by SCr and UO. [file 13613_2017_251_MOESM6_ESM.pdf]

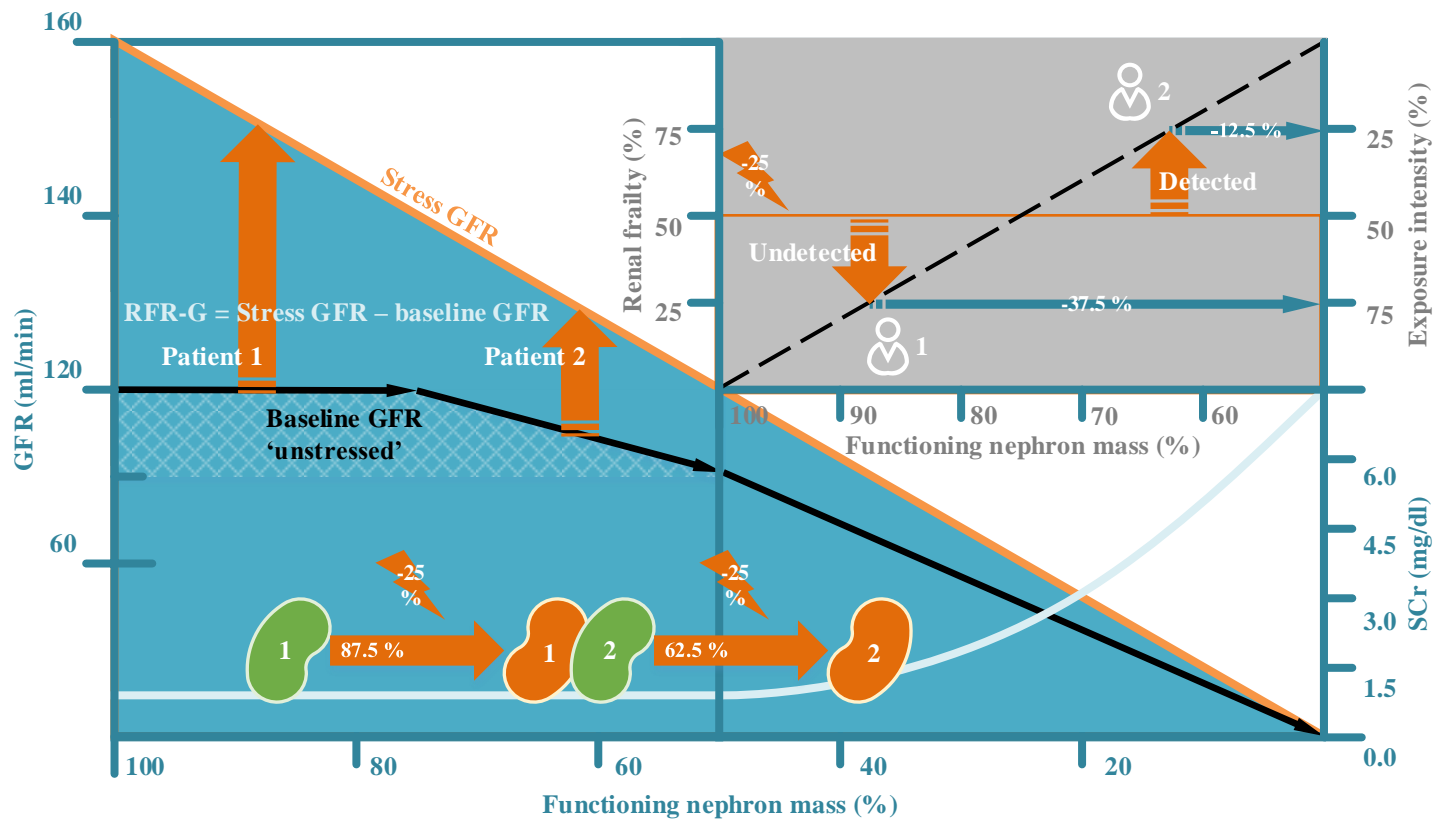

Supplement: Supplementary file 8 — Additional file 8: Figure S6. Renal functional reserve of the glomerular function and functioning nephron mass. [file 13613_2017_251_MOESM8_ESM.pdf]
