# Supplementary material for: Diagnosis of cardiac surgery-associated acute kidney injury: differential roles of creatinine, chitinase 3-like protein 1 and neutrophil gelatinase-associated lipocalin: a prospective cohort study
Source: Ann Intensive Care. 2017 Mar 1;7:24. doi: 10.1186/s13613-017-0251-z (PMC5332341; doi:10.1186/s13613-017-0251-z)
Supplement: Supplementary file 5 — Additional file 5. Table S1: Data collected in the clinical research file. Table S2: Dilution of serum and urine samples for the initial measurement of CHI3L1 by ELISA. Table S3: Characteristics of the patients and procedures peri-operatively. Table S4A: Biomarker performances at t0 for prediction of AKI. Table S4B: Biomarker performances at t1 for prediction of AKI. Table S4C: Biomarker performances at t2 for prediction of AKI. Table S4D: Biomarker performances at t3 for prediction of AKI. [file 13613_2017_251_MOESM5_ESM.pdf]

ADDITIONAL FILE 5

SUPPLEMENTAL TABLES

Table S1 Data collected in the clinical research file

I. Patient information	
A. Study identification	A / □□□
B. Date of birth	□□ / □□ / □□□□
C. Sex	□ Male or □ Female
D. Race	□ Caucasian or □ Black
E. Smoking status	□ Current smoker or □ Ex-smoker or □ Non-smoker
♦ Smoking history: cumulative number of pack-years[a]	▬ Textbox
F. Alcohol use	□ Yes or □ No
♦ Quantity of alcohol intake	▬ Textbox
G. Weight (kg)	▬ Textbox
H. Length (cm)	▬ Textbox
I. BMI	▬ Textbox
II. Medical data	
A. Medical history	
1. NYHA class	□ I or □ II or □ III or □ IV
2. DM	□ Type 1 or □ Type 2 or □ No DM
♦ Therapy for DM	□ Insulin therapy or □ Therapy per os
3. Other comorbidities	▬ Textbox
4. Pre-operative medication list	▬ Textbox
B. Admission diagnosis	
1. Date of hospital admission	□□ / □□ / □□□□
♦ Diagnosis at hospital admission	▬ Textbox
2. Date of ICU admission	□□ / □□ / □□□□
♦ Diagnosis at ICU admission	▬ Textbox
C. Baseline registrations	

1. Blood pressure (mm Hg; highest)	
i. Systolic / Diastolic	▬ Textbox
ii. Mean	▬ Textbox
2. Heart rhythm	□ Atrial fibrillation or □ Normal sinus rhythm
3. Heart rate in normal sinus rhythm (bpm)	▬ Textbox
4. Ejection fraction (%)	
i. Pre-operative	▬ Textbox
ii. Post-operative	▬ Textbox
5. Fractional shortening (%)	
i. Pre-operative	▬ Textbox
ii. Post-operative	▬ Textbox
6. Reference SCr	▬ Textbox
7. Reference $eGFR_{CKD-EPI}$	▬ Textbox
8. Medication	
i. Statins	□ Yes or □ No
ii. ACE inhibitors	□ Yes or □ No
iii. ARBs	□ Yes or □ No
iv. Diuretics	□ Yes or □ No
v. NSAIDs	□ Yes or □ No
vi. Corticosteroids	□ Yes or □ No
vii. Tacrolimus	□ Yes or □ No
viii. Cyclosporine	□ Yes or □ No
ix. Aminoglycosides	□ Yes or □ No
x. Iodinated contrast pre-operatively	□ Yes or □ No
♦ Date	□□ / □□ / □□□□
xi. Corticosteroids intra-operatively	□ Yes or □ No
D. Index surgical procedure	
1. Euroscore	▬ Textbox
2. Type of cardiac surgical procedure	▬ Textbox

3. Duration of surgery (h)	▬ Textbox
4. IABP peri-operatively	□ Yes or □ No
5. ECC data	
i. Mean blood pressure on pump	▬ Textbox
ii. Diuretics on pump	□ Yes or □ No
iii. Diuresis on pump (ml)	▬ Textbox
iv. Priming volume of pump (ml)	▬ Textbox
v. Haematocrit (%)	
♦ Before pump	▬ Textbox
♦ After pump	▬ Textbox
vi. SCr (mg/dl)	
♦ Before pump	▬ Textbox
♦ After pump	▬ Textbox
vii. Duration of ECC (min)	▬ Textbox
viii. Duration of aortic clamp (min)	▬ Textbox
ix. Duration of ischemia (min)	▬ Textbox
E. Study follow-up (to be filled in for $d_{surgery}$, $d_{1post\text{-}op}$ and $d_{2post\text{-}op}$)	
1. RRT	□ Yes or □ No
♦ Date	□□ / □□ / □□□□
2. SOFA score	▬ Textbox
3. WBC count (10^3/µl)	▬ Textbox
4. Serum CRP (mg/l)	▬ Textbox
5. Fluid balance	▬ Textbox
♦ Total fluid IN (ml)	▬ Textbox
♦ Total fluid OUT (ml)	▬ Textbox
6. Transfusions	
i. Whole blood	□ Yes or □ No
♦ Number of units transfused	▬ Textbox

ii. Plasma	□ Yes or □ No
♦ Number of units transfused	▬ Textbox
iii. Platelets	□ Yes or □ No
♦ Number of units transfused	▬ Textbox
7. Medication	
i. Milrinone (PDE3 inhibitor)	□ Yes or □ No
ii. Vasopressor(s)	□ Yes or □ No
♦ Generic name(s)	▬ Textbox
iii. Antibiotic(s)	□ Yes or □ No
♦ Generic name(s)	▬ Textbox
iv. Statins	□ Yes or □ No
v. ACE inhibitors	□ Yes or □ No
vi. ARBs	□ Yes or □ No
vii. Diuretics	□ Yes or □ No
viii. Tacrolimus	□ Yes or □ No
ix. Cyclosporine	□ Yes or □ No
x. Iodinated contrast post-operatively	□ Yes or □ No

[a]A pack-year is defined as twenty cigarettes smoked every day for one year

ACE angiotensin-converting enzyme, *ARB* angiotensin-II receptor blocker, *BMI* body mass index, *bpm* beats per minute, *CKD-EPI* Chronic Kidney Disease Epidemiology Collaboration, *CRP* C-reactive protein, *d* day, *DM* diabetes mellitus, *ECC* extracorporeal circulation, *eGFR* estimated glomerular filtration rate, *EuroSCORE* European System for Cardiac Operative Risk Evaluation, *h* hour, *IABP* intra-aortic balloon pump, *ICU* intensive care unit, *min* minute, *NSAID* non-steroidal anti-inflammatory drug, *NYHA* New York Heart Association, *PDE3* phosphodiesterase 3, *post-op* post-operatively, *RRT* renal replacement therapy, *SCr* serum creatinine, *SOFA* Sepsis-related Organ Failure Assessment, *WBC* white blood cell

Table S2 Dilution of serum and urine samples for the initial measurement of CHI3L1 by ELISA

Time point of the study	Estimated dilution for serum sample	Estimated dilution for urine sample
t0	1/100	1/2
t1	1/100	1/2
t2	1/200	1/4
t3	1/200	1/4
t4	1/200	1/4
t5	1/500	1/4
t6	1/500	1/4
t7	1/500	1/4

CHI3L1 chitinase 3-like protein 1, *ELISA* enzyme-linked immunosorbent assay

Table S3 Characteristics of the patients and procedures peri-operatively

Time	Day of surgery				First post-operative day				Second post-operative day			
	All patients	AKI stage ≥ 1[a] within 48 h	No AKI[a] within 48 h	P value	All patients	AKI stage ≥ 1[a] within 48 h	No AKI[a] within 48 h	P value	All patients	AKI stage ≥ 1[a] within 48 h	No AKI[a] within 48 h	P value
No. (%) [95 % CI]	203 (100) [98.1-100]	95 (46.8) [40.1-53.7]	108 (53.2) [46.3-59.9]		203 (100) [98.1-100]	95 (46.8) [40.1-53.7]	108 (53.2) [46.3-59.9]		203 (100) [98.1-100]	95 (46.8) [40.1-53.7]	108 (53.2) [46.3-59.9]	
Characteristic (IQR)												
SOFA score	9 (8-10)	9 (8-10)	8 (7-9)	< 0.001	5 (3-7)	6 (4-8)	4 (3-6)	< 0.001	3 (2-4)	4 (3-6)	2 (1-3)	< 0.001
WBC count – 10^3/µl	10.7 (8.4-12.9)	10.6 (8.0-13.4)	10.7 (8.5-12.9)	0.958	12.4 (9.9-14.9)	12.7 (11.0-15.8)	11.5 (9.3-14.9)	0.032	12.0 (10.0-14.8)	12.9 (10.3-15.9)	11.5 (9.7-14.4)	0.012
Serum CRP – mg/l	9.5 (4.0-18.0)	9.8 (5.0-16.5)	9.3 (3.4-18.0)	0.591	67.0 (37.6-118.7)	89.0 (45.5-137.4)	56.0 (36.0-95.8)	0.001	198.4 (127.3-246.3)	205.0 (156.8-265.4)	143.6 (117.6-230.8)	0.043
Fluid balance – ml	1014 (598-1460)	1269 (827-1613)	797 (432-1235)	< 0.001	568 (226$_{neg}$-1518)	1105 (190-1977)	291 (512$_{neg}$-1084)	< 0.001	299 (597$_{neg}$-934)	549 (186$_{neg}$-1206)	26 (880$_{neg}$-781)	0.004
Procedure												

ADDITIONAL FILE 5

Transfusion(s) – no. (%) [95 % CI]												
Whole blood	37 (18.2) [13.5-24.1]	22 (23.2) [15.8-32.6]	15 (13.9) [8.6-21.7]	0.102	13 (6.4) [3.8-10.6]	8 (8.4) [4.3-15.7]	5 (4.6) [2.0-10.4]	0.390	11 (5.4) [3.1-9.4]	7 (7.4) [3.6-14.4]	4 (3.7) [1.4-9.1]	0.354
Plasma	14 (6.9) [4.2-11.2]	6 (6.3) [2.9-13.1]	8 (7.4) [3.8-13.9]	0.789	3 (1.5) [0.5-4.3]	2 (2.1) [0.6-7.4]	1 (0.9) [0.2-5.1]	0.600	3 (1.5) [0.5-4.3]	3 (3.2) [1.1-8.9]	0 (0.0) [0.0-3.4]	0.101
Platelets	11 (5.4) [3.1-9.4]	8 (8.4) [4.3-15.7]	3 (2.8) [0.9-7.9]	0.118	1 (0.5) [0.1-2.7]	1 (1.1) [0.2-5.7]	0 (0.0) [0.0-3.4]	0.468	1 (0.5) [0.1-2.7]	1 (1.1) [0.2-5.7]	0 (0.0) [0.0-3.4]	0.468
No. of units transfused (IQR)												
Whole blood	2.0 (1.0-3.0)	2.0 (1.0-3.5)	2.0 (1.0-2.0)	0.531	1.0 (1.0-2.0)	1.0 (1.0-2.0)	1.0 (1.0-2.0)	1.000	1.0 (1.0-2.0)	1.0 (1.0-3.0)	1.0 (1.0-1.0)	0.315
Plasma	3.0 (2.0-4.3)	4.5 (3.8-5.3)	2.0 (2.0-2.8)	0.001	1.0 (1.0-)	1.5 (1.0-)	1.0 (1.0-1.0)	1.000	3.0 (1.0-)	3.0 (1.0-)	NA	NA
Platelets	9.0 (6.0-10.0)	8.5 (6.5-10.0)	10.0 (1.0-)	0.921	1.0 (1.0-1.0)	1.0 (1.0-1.0)	NA	NA	10.0 (10.0-10.0)	10.0 (10.0-10.0)	NA	NA
Medication – no. (%) [95 % CI]												
Vasopressors	98 (48.3)	49 (51.6)	49 (45.4)	0.401	92 (45.3)	48 (50.5)	44 (40.7)	0.203	29 (14.3)	20 (21.1)	9 (8.3)	0.015

	[41.5-55.1]	[41.7-61.4]	[36.3-54.8]		[38.6-52.2]	[40.6-60.4]	[31.9-50.2]		[10.1-19.8]	[14.1-30.3]	[4.4-15.1]	
Milrinone (PDE3 inhibitor)	19 (9.4) [6.1-14.2]	14 (14.7) [9.0-23.2]	5 (4.6) [2.0-10.4]	0.016	21 (10.3) [6.9-15.3]	15 (15.8) [9.8-24.4]	6 (5.6) [2.6-11.6]	0.021	14 (6.9) [4.2-11.2]	10 (10.5) [5.8-18.3]	4 (3.7) [1.4-9.1]	0.093
Statins	4 (2.0) [0.8-5.0]	1 (1.1) [0.2-5.7]	3 (2.8) [0.9-7.9]	0.624	93 (45.8) [39.1-52.7]	44 (46.3) [36.6-56.3]	49 (45.4) [36.3-54.8]	1.000	107 (52.7) [45.9-59.5]	46 (48.4) [38.6-58.3]	61 (56.5) [47.1-65.5]	0.263
ACE inhibitors	1 (0.5) [0.1-2.7]	0 (0.0) [0.0-3.9]	1 (0.9) [0.2-5.1]	1.000	20 (9.9) [6.5-14.7]	8 (8.4) [4.3-15.7]	12 (11.1) [6.5-18.4]	0.639	29 (14.3) [10.1-19.8]	12 (12.6) [7.4-20.8]	17 (15.7) [10.1-23.8]	0.554
ARBs	0 (0.0) [0.0-1.9]	0 (0.0) [0.0-3.9]	0 (0.0) [0.0-3.4]	NA	3 (1.5) [0.5-4.3]	1 (1.1) [0.2-5.7]	2 (1.9) [0.5-6.5]	1.000	3 (1.5) [0.5-4.3]	1 (1.1) [0.2-5.7]	2 (1.9) [0.5-6.5]	1.000
Diuretics	12 (5.9) [3.4-10.0]	7 (7.4) [3.6-14.4]	5 (4.6) [2.0-10.4]	0.553	120 (59.1) [52.2-65.6]	73 (76.8) [67.4-84.2]	47 (43.5) [34.5-52.9]	< 0.001	93 (45.8) [39.1-52.7]	55 (57.9) [47.8-67.3]	38 (35.2) [26.8-44.6]	0.002
Tacrolimus	0 (0.0) [0.0-1.9]	0 (0.0) [0.0-3.9]	0 (0.0) [0.0-3.4]	NA	0 (0.0) [0.0-1.9]	0 (0.0) [0.0-3.9]	0 (0.0) [0.0-3.4]	NA	0 (0.0) [0.0-1.9]	0 (0.0) [0.0-3.9]	0 (0.0) [0.0-3.4]	NA
Cyclosporine	0 (0.0) [0.0-1.9]	0 (0.0) [0.0-3.9]	0 (0.0) [0.0-3.4]	NA	2 (1.0) [0.3-3.5]	2 (2.1) [0.6-7.4]	0 (0.0) [0.0-3.4]	0.218	2 (1.0) [0.3-3.5]	2 (2.1) [0.6-7.4]	0 (0.0) [0.0-3.4]	0.218
Aminoglycosides	3 (1.5) [0.5-4.3]	1 (1.1) [0.2-5.7]	2 (1.9) [0.5-6.5]	1.000	3 (1.5) [0.5-4.3]	1 (1.1) [0.2-5.7]	2 (1.9) [0.5-6.5]	1.000	2 (1.0) [0.3-3.5]	1 (1.1) [0.2-5.7]	1 (0.9) [0.2-5.1]	1.000
Iodinated contrast post-operative	0 (0.0) [0.0-1.9]	0 (0.0) [0.0-3.9]	0 (0.0) [0.0-3.4]	NA	1 (0.5) [0.1-2.7]	1 (1.1) [0.2-5.7]	0 (0.0) [0.0-3.4]	0.468	2 (1.0) [0.3-3.5]	1 (1.1) [0.2-5.7]	1 (0.9) [0.2-5.1]	1.000

ADDITIONAL FILE 5

[a]KDIGO definitions for the diagnosis and staging of AKI, which are based on SCr and UO

ACE angiotensin converting enzyme, *AKI* acute kidney injury, *ARB* angiotensin-II receptor blocker, *CI* confidence interval, *CRP* C-reactive protein, *h* hour, *IQR* interquartile range, *KDIGO* Kidney Disease: Improving Global Outcomes, *no.* number, *PDE3* phosphodiesterase 3, *SCr* serum creatinine, *SOFA* Sepsis-related Organ Failure Assessment, *UO* urine output, *WBC* white blood cell

Table S4A Biomarker performances at t0 for prediction of AKI

Endpoint	AKI stage ≥ 2[a] within 12 h after t1			AKI stage ≥ 1[a] within 48 h after t1		
	AUC-ROC	Lower limit of 95 % CI	Upper limit of 95 % CI	AUC-ROC	Lower limit of 95 % CI	Upper limit of 95 % CI
[SCHI3L1]•[UCHI3L1]	0.595	0.524	0.663	0.599	0.526	0.668
[SCr]•[SCHI3L1]	0.580	0.510	0.648	0.705	0.637	0.768
[SCr]•[UCHI3L1]	0.669	0.599	0.733	0.582	0.510	0.652
SCHI3L1	0.508	0.438	0.578	0.660	0.590	0.725
UCHI3L1/UCr	0.530	0.459	0.601	0.518	0.445	0.589
UCHI3L1	0.578	0.507	0.647	0.532	0.459	0.603
SCr	0.696	0.629	0.758	0.709	0.641	0.771

[a]KDIGO definitions for the diagnosis and staging of AKI, which are based on SCr and UO

AKI acute kidney injury, *AUC-ROC* area under the receiver-operating characteristics curve, *CI* confidence interval, *h* hour, *KDIGO* Kidney Disease | Improving Global Outcomes, *SCHI3L1* serum chitinase 3-like protein 1, *SCr* serum creatinine, *t0* time after the induction of anaesthesia and before the start of surgery, *t1* time of intensive care unit admission, *UCHI3L1* urinary chitinase 3-like protein 1, *UCr* urinary creatinine, *UO* urine output

Table S4B Biomarker performances at t1 for prediction of AKI

Endpoint	AKI stage ≥ 2[a] within 12 h after t1			AKI stage ≥ 1[a] within 48 h after t1		
	AUC-ROC	Lower limit of 95 % CI	Upper limit of 95 % CI	AUC-ROC	Lower limit of 95 % CI	Upper limit of 95 % CI
ΔSCr	0.833	0.776	0.881	0.504	0.433	0.575
[SCHI3L1]•[UNGAL]	0.659	0.590	0.724	0.646	0.575	0.713
[SCHI3L1]•[UCHI3L1]	0.633	0.562	0.699	0.621	0.549	0.690
[UCHI3L1]•[UNGAL]	0.615	0.544	0.682	0.570	0.497	0.640
[SCr]•[SCHI3L1]	0.611	0.541	0.678	0.725	0.658	0.786
[SCr]•[UNGAL]	0.718	0.651	0.779	0.661	0.590	0.726
[SCr]•[UCHI3L1]	0.665	0.596	0.730	0.598	0.526	0.667
SCHI3L1	0.505	0.435	0.575	0.671	0.601	0.735
UNGAL/UCr	0.648	0.578	0.713	0.599	0.528	0.668
UNGAL	0.650	0.581	0.716	0.583	0.511	0.653
UCHI3L1/UCr	0.631	0.561	0.698	0.557	0.484	0.628
UCHI3L1	0.621	0.550	0.688	0.556	0.484	0.627
SCr	0.780	0.718	0.834	0.735	0.669	0.794

[a]KDIGO definitions for the diagnosis and staging of AKI, which are based on SCr and UO

AKI acute kidney injury, *AUC-ROC* area under the receiver-operating characteristics curve, *CI* confidence interval, *ΔSCr* represents $\Delta SCr_{t1\text{-}t0}$, which is the absolute change in SCr between SCr_{t1} and SCr_{t0}, *h* hour, *KDIGO* Kidney Disease | Improving Global Outcomes, *SCHI3L1* serum chitinase 3-like protein 1, *SCr* serum creatinine, *t0* time after the induction of anaesthesia and before the start of surgery, *t1* time of intensive care unit admission, *UCHI3L1* urinary chitinase 3-like protein 1, *UCr* urinary creatinine, *UNGAL* urinary neutrophil gelatinase-associated lipocalin, *UO* urine output

Table S4C Biomarker performances at t2 for prediction of AKI

Endpoint	AKI stage ≥ 2[a] within 12 h after t1			AKI stage ≥ 1[a] within 48 h after t1		
	AUC-ROC	Lower limit of 95 % CI	Upper limit of 95 % CI	AUC-ROC	Lower limit of 95 % CI	Upper limit of 95 % CI
ΔSCr	0.915	0.840	0.991	0.556	0.471	0.641
[SCHI3L1]•[UCHI3L1]	0.773	0.708	0.829	0.643	0.571	0.711
[SCr]•[SCHI3L1]	0.725	0.658	0.786	0.695	0.625	0.760
[SCr]•[UCHI3L1]	0.746	0.680	0.805	0.651	0.579	0.718
SCHI3L1	0.628	0.557	0.695	0.649	0.577	0.716
UCHI3L1/UCr	0.662	0.593	0.726	0.574	0.502	0.644
UCHI3L1	0.686	0.617	0.748	0.575	0.503	0.645
SCr	0.821	0.760	0.871	0.761	0.694	0.819

[a]KDIGO definitions for the diagnosis and staging of AKI, which are based on SCr and UO

AKI acute kidney injury, *AUC-ROC* area under the receiver-operating characteristics curve, *CI* confidence interval, *ΔSCr* represents $\Delta SCr_{t2\text{-}t0}$, which is the absolute change in SCr between SCr_{t2} and SCr_{t0}, *h* hour, *KDIGO* Kidney Disease | Improving Global Outcomes, *SCHI3L1* serum chitinase 3-like protein 1, *SCr* serum creatinine, *t0* time after the induction of anaesthesia and before the start of surgery, *t1* time of intensive care unit admission, *t2* 2 hours after intensive care unit admission, *UCHI3L1* urinary chitinase 3-like protein 1, *UCr* urinary creatinine, *UO* urine output

Table S4D Biomarker performances at t3 for prediction of AKI

Endpoint	AKI stage ≥ 2[a] within 12 h after t1			AKI stage ≥ 1[a] within 48 h after t1		
	AUC-ROC	Lower limit of 95 % CI	Upper limit of 95 % CI	AUC-ROC	Lower limit of 95 % CI	Upper limit of 95 % CI
ΔSCr	0.938	0.860	1.000	0.643	0.562	0.724
[SCHI3L1]•[UNGAL]	0.774	0.710	0.830	0.665	0.594	0.731
[SCHI3L1]•[UCHI3L1]	0.758	0.692	0.816	0.684	0.613	0.749
[UCHI3L1]•[UNGAL]	0.678	0.610	0.741	0.633	0.563	0.700
[SCr]•[SCHI3L1]	0.814	0.754	0.866	0.723	0.654	0.785
[SCr]•[UNGAL]	0.728	0.660	0.788	0.673	0.602	0.738
[SCr]•[UCHI3L1]	0.754	0.688	0.812	0.725	0.656	0.786
SCHI3L1	0.720	0.653	0.781	0.664	0.593	0.730
UNGAL/UCr	0.649	0.579	0.713	0.600	0.529	0.669
UNGAL	0.656	0.587	0.720	0.612	0.541	0.679
UCHI3L1/UCr	0.653	0.584	0.718	0.617	0.546	0.685
UCHI3L1	0.678	0.610	0.741	0.649	0.578	0.715
SCr	0.857	0.801	0.902	0.792	0.728	0.847

[a]KDIGO definitions for the diagnosis and staging of AKI, which are based on SCr and UO

AKI acute kidney injury, *AUC-ROC* area under the receiver-operating characteristics curve, *CI* confidence interval, *ΔSCr* represents ΔSCr_{t3-t0}, which is the absolute change in SCr between SCr_{t3} and SCr_{t0}, *h* hour, *KDIGO* Kidney Disease | Improving Global Outcomes, *SCHI3L1* serum chitinase 3-like protein 1, *SCr* serum creatinine, *t0* time after the induction of anaesthesia and before the start of surgery, *t1* time of intensive care unit admission, *t3* 4 hours after intensive care unit admission, *UCHI3L1* urinary chitinase 3-like protein 1, *UCr* urinary creatinine, *UNGAL* urinary neutrophil gelatinase-associated lipocalin, *UO* urine output

LEGENDS OF SUPPLEMENTAL FIGURES

Additional file 1: Fig. S1 STROBE statement – checklist of items that should be included in the reports of cohort studies (1)

No. number, *STROBE* STrengthening the Reporting of OBservational studies in Epidemiology

Additional file 2: Fig. S2 KDIGO definition and classification of AKI (2)

[a]For staging purposes, patients should be staged according to the criterion or criteria that give(s) them the highest stage.

AKI acute kidney injury, *d* day, *h* hour, *KDIGO* Kidney Disease | Improving Global Outcomes, *RRT* renal replacement therapy, *SCr* serum creatinine, *UO* urine output

Additional file 3: Fig. S3A Study course and sample collection times in a fictional morning patient

d day, *h* hour, *MAKE* major adverse kidney event, *mo* month, *post-op* post-operatively, *SCr* serum creatinine, *t* time, *y* year

Additional file 4: Fig. S3B Study course and sample collection times in a fictional afternoon patient

d day, *h* hour, *MAKE* major adverse kidney event, *mo* month, *post-op* post-operatively, *SCr* serum creatinine, *t* time, *y* year

Additional file 6: Fig. S4 Dissociation of the KDIGO definitions for the diagnosis and staging of AKI by SCr and UO

AKI acute kidney injury, *h* hour, *KDIGO* Kidney Disease | Improving Global Outcomes, *SCr* serum creatinine, *UO* urine output

Additional file 7: Fig. S5 Flow of patients over different diagnostic windows for AKI stage ≥ 2

[a]Planned ≥ 4 h in advance

[b]KDIGO definitions for the diagnosis and staging of AKI, which are based on SCr and UO (2)

[c]KDOQI definitions for the diagnosis and staging of CKD (3)

[d]≤ 3 mo before

AKI acute kidney injury, *CKD* chronic kidney disease, *d* day, *h* hour, *ICU* intensive care unit, *KDIGO* Kidney Disease | Improving Global Outcomes, *KDOQI* Kidney Disease Outcomes Quality Initiative, *mo* month, *No.* number, *Sat* Saturday, *SCr* serum creatinine, *Sun* Sunday, *UO* urine output, *y* year

Additional file 8: Fig. S6 Renal functional reserve of the glomerular function and functioning nephron mass

Suppose that when all nephrons are functioning baseline GFR is 120 ml/min and that when stressed this GFR can reach 160 ml/min, indicating a RFR-G of 40 ml/min. With 87.5 % functioning nephrons, patient 1 will maintain a baseline GFR of 120 ml/min and reach a lower stress GFR of 150 ml/min. Patient 2 with 62.5 % functioning nephrons will maintain a lower, but still normal, baseline GFR of 105 ml/min and reach a lower stress GFR of 125 ml/min. An exposure that leads to 25 % loss of functioning nephron mass will lead to an increased SCr concentration in patient 2, but will remain undetected in patient 1. Note: the population variability of the RFR-G response is not known; represented values are only illustrative.

ADDITIONAL FILE 5

GFR glomerular filtration rate, *RFR-G* renal functional reserve of the glomerular function, *SCr* serum creatinine

REFERENCES

1. Vandenbroucke JP, von Elm E, Altman DG, Gotzsche PC, Mulrow CD, Pocock SJ, et al. Strengthening the reporting of observational studies in epidemiology (STROBE): explanation and elaboration. Plos Med. 2007;4:1628-54.

2. Kidney Disease: Improving Global Outcomes (KDIGO) acute kidney injury work group. KDIGO clinical practice guideline for acute kidney injury. Kidney International Suppl. 2012;2:1-138.

3. Eknoyan G, Levin NW. KDOQI clinical practice guidelines for chronic kidney disease: evaluation, classification, and stratification - foreword. Am J Kidney Dis. 2002;39:S14-S266.
