# Supplementary material for: Diagnosis of cardiac surgery-associated acute kidney injury: differential roles of creatinine, chitinase 3-like protein 1 and neutrophil gelatinase-associated lipocalin: a prospective cohort study
Source: Ann Intensive Care. 2017 Mar 1;7:24. doi: 10.1186/s13613-017-0251-z (PMC5332341; doi:10.1186/s13613-017-0251-z)
Supplement: Supplementary file 7 — Additional file 7: Figure S5. Flow of patients over different diagnostic windows for AKI stage ≥2. [file 13613_2017_251_MOESM7_ESM.pdf]

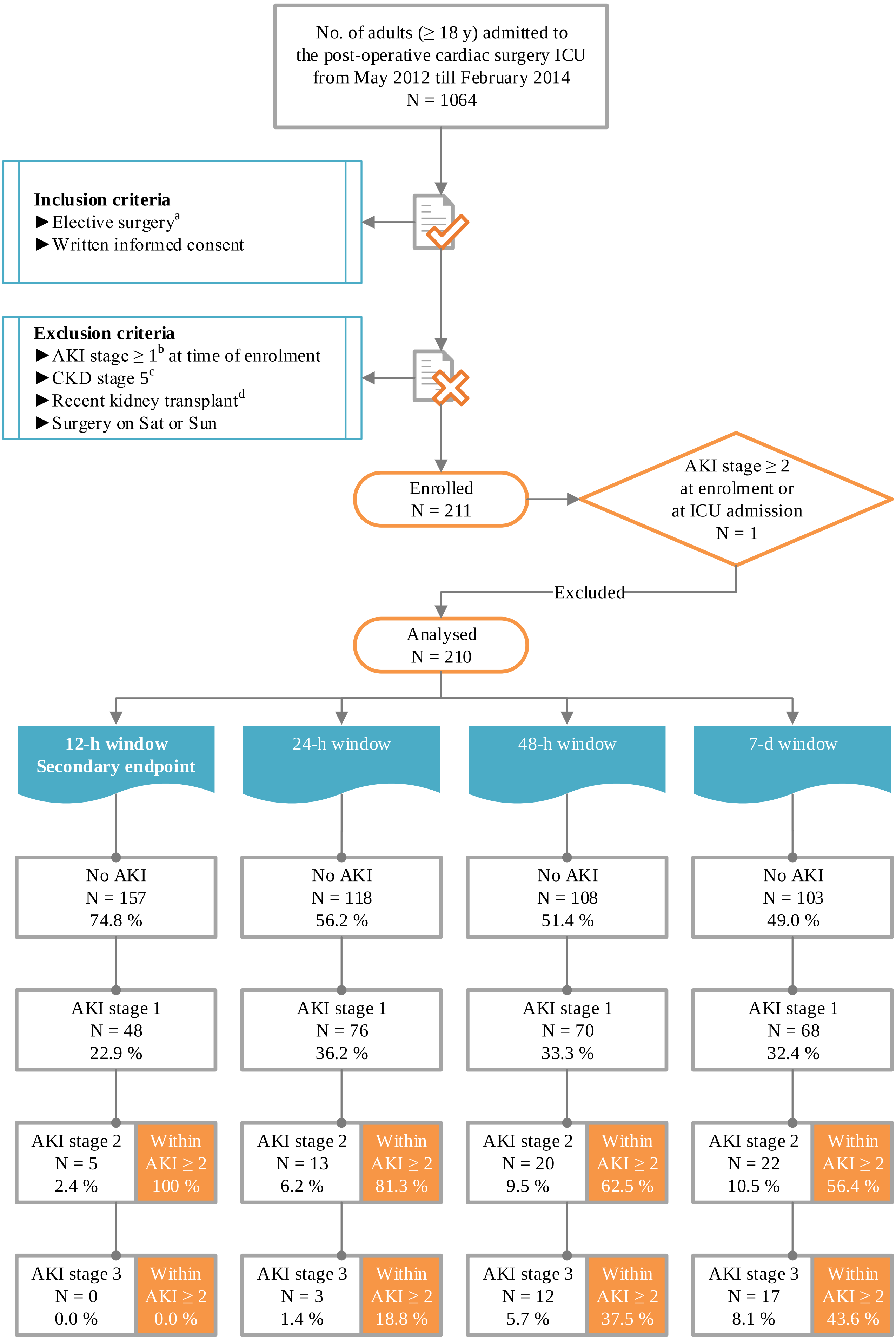
No. of adults (≥ 18 y) admitted to the post-operative cardiac surgery ICU from May 2012 till February 2014
N = 1064
Inclusion criteria
▶ Elective surgery[a]
▶ Written informed consent
Exclusion criteria
▶ AKI stage ≥ 1[b] at time of enrolment
▶ CKD stage 5[c]
▶ Recent kidney transplant[d]
▶ Surgery on Sat or Sun
Enrolled
N = 211
AKI stage ≥ 2 at enrolment or at ICU admission
N = 1
Excluded
Analysed
N = 210
12-h window Secondary endpoint
24-h window
48-h window
7-d window
No AKI N = 157 74.8 %
No AKI N = 118 56.2 %
No AKI N = 108 51.4 %
No AKI N = 103 49.0 %
AKI stage 1 N = 48 22.9 %
AKI stage 1 N = 76 36.2 %
AKI stage 1 N = 70 33.3 %
AKI stage 1 N = 68 32.4 %
AKI stage 2 N = 5 2.4 %
Within AKI ≥ 2 100 %
AKI stage 2 N = 13 6.2 %
Within AKI ≥ 2 81.3 %
AKI stage 2 N = 20 9.5 %
Within AKI ≥ 2 62.5 %
AKI stage 2 N = 22 10.5 %
Within AKI ≥ 2 56.4 %
AKI stage 3 N = 0 0.0 %
Within AKI ≥ 2 0.0 %
AKI stage 3 N = 3 1.4 %
Within AKI ≥ 2 18.8 %
AKI stage 3 N = 12 5.7 %
Within AKI ≥ 2 37.5 %
AKI stage 3 N = 17 8.1 %
Within AKI ≥ 2 43.6 %
